# Activation of Pro-survival CaMK4β/CREB and Pro-death MST1 signaling at early and late times during a mouse model of prion disease

**DOI:** 10.1186/1743-422X-11-160

**Published:** 2014-09-02

**Authors:** Rory H Shott, Anna Majer, Kathy L Frost, Stephanie A Booth, Luis M Schang

**Affiliations:** Department of Biochemistry and Centre for Prions and Protein Folding Diseases (CPPFD), University of Alberta, Edmonton, AB Canada; Li Ka Shing Institute of Virology, University of Alberta, Edmonton, AB Canada; Molecular PathoBiology, National Microbiology Laboratory, Public Health Agency of Canada, Winnipeg, MB Canada; Department of Medical Microbiology and Infectious Diseases, University of Manitoba, Winnipeg, MB Canada

**Keywords:** Prion disease, Kinomics, Protein kinase, Multiplex Western blots, CaMK4β, CREB, MST1

## Abstract

**Background:**

The signaling pathways most critical to prion disease pathogenesis are as yet incompletely characterized. We have developed a kinomics approach to identify signaling pathways that are dysregulated during prion pathogenesis. The approach is sensitive and specific enough to detect signaling pathways dysregulated in a simple in vitro model of prion pathogenesis. Here, we used this approach to identify signaling pathways dysregulated during prion pathogenesis in vivo.

**Methods:**

Mice intraperitoneally infected with scrapie (strain RML) were euthanized at 70, 90, 110, 130 days post-infection (dpi) or at terminal stages of disease (155–190 dpi). The levels of 139 protein kinases in brainstem-cerebellum homogenates were analyzed by multiplex Western blots, followed by hierarchical clustering and analyses of activation states.

**Results:**

Hierarchical and functional clustering identified CaMK4β and MST1 signaling pathways as potentially dysregulated. Targeted analyses revealed that CaMK4β and its downstream substrate CREB, which promotes neuronal survival, were activated at 70 and 90 dpi in cortical, subcortical and brainstem-cerebellum homogenates from scrapie-infected mice. The activation levels of CaMK4β/CREB signaling returned to those in mock-infected mice at 110 dpi, whereas MST1, which promotes neuronal death, became activated at 130 dpi.

**Conclusion:**

Pro-survival CaMK4β/CREB signaling is activated in mouse scrapie at earlier times and later inhibited, whereas pro-death MST1 signaling is activated at these later times.

**Electronic supplementary material:**

The online version of this article (doi:10.1186/1743-422X-11-160) contains supplementary material, which is available to authorized users.

## Background

Prion diseases are a family of invariably lethal chronic neurodegenerative diseases that affect humans (kuru; Creutzfeldt-Jakob disease, CJD; Gerstmann-Sträussler-Scheinker disease, GSS; fatal familial insomnia, FFI), and other species such as cattle (bovine spongiform encephalopathy, BSE), goat, sheep (scrapie), deer, elk and moose (chronic wasting disease, CWD) [1, 2]. Human prion diseases can be infectious (acquired), inherited (genetic), or sporadic. The latter are the most common, accounting for approximately 85% of cases [3]. Whereas the inherited and acquired cases may be suspected, from the risk facts, before the onset of clinical symptom, the sporadic cases can only be diagnosed after the onset of clinical symptoms [4].

The neuropathology of prion diseases is characterized by gliosis, spongiform degeneration, and neuronal death. The conversion of the cellular prion protein (PrP^C^) into its pathological conformation (PrP^Sc^) is essential for pathogenesis. Neuronal death and disease progression were prevented in scrapie-infected mice by conditional ablation of PrP^C^ at the time when PrP^Sc^ was first detected [5]. Inhibition of PrP^C^ conversion to PrP^Sc^ is thus a validated target for therapeutic intervention. Many compounds have been identified to inhibit PrP conversion or the accumulation of PrP^Sc^ in vitro [6, 7]. Only one, however, the diphenyl pyrazole derivative anle138b [3-(1,3-benzodioxol-5-yl)-5-(3-bromophenyl)-1H-pyrazole], prolonged survival of scrapie-infected mice when treatment was started after the onset of clinical signs of disease [8]. Unfortunately, none affected survival of patients with CJD, GSS, or FFI [9–18]. Another compound that prolonged survival of scrapie-infected mice after the onset of clinical disease is the calcineurin/ protein phosphatase 3 inhibitor FK506. FK506, however, did not affect the levels of PrP^C^ or the accumulation of PrP^Sc^ [19], indicating that FK506 acts downstream from the accumulation of PrP^Sc^. As FK506 is a known (calcineurin) signaling inhibitor, these results suggest that dysregulated signaling downstream of PrP conversion is an alternative therapeutic target against prion diseases.

Protein kinases (and phosphatases) modulate by reversible phosphorylation the function, localization, or activities of approximately one-third of cellular proteins [20]. Protein kinases are therefore critical regulators of signal transduction. Their dysregulation is implicated in the pathogenesis of many chronic diseases, including neurodegenerative diseases such as Alzheimer’s and Parkinson’s [21–23]. Consequently, protein kinases are major therapeutic targets. It is estimated that up to 30% of the research and development budget of the pharmaceutical industry is invested in protein kinase inhibitors [24, 25]. For example, protein kinase inhibitors are the largest group of new cancer therapeutics [26]. Thirty-one protein kinase inhibitors are in clinical use, over 500 are involved in approximately 2,700 clinical trials, and thousands more are in various stages of pre-clinical development ( [26–28] and summary of [29]). Protein kinase inhibitors therefore constitute a rapidly growing group of clinical drugs that have the potential to considerably impact treatment of chronic diseases.

Considering the critical roles that protein kinases play in the pathogenesis of other chronic neurodegenerative diseases, it is not surprising that they also participate in the pathogenesis of prion diseases. For example, the activation of vascular endothelial growth factor receptor (VEGFR) inhibited death of cultured neurons treated with the neurotoxic prion peptide PrP106-126 [30]. Death of PrP106-126-treated cultured neurons was also inhibited by Abelson leukemia oncogene cellular homolog (c-Abl) knockdown [31] and treatment with the glycogen synthase kinase 3 (GSK3) inhibitor lithium [32]. Scrapie-infected mice treated with the protein kinase R-like endoplasmic reticulum kinase (PERK) inhibitor GSK2606414 survived longer than vehicle-treated mice [33]. The phosphoinositide-dependent kinase 1 (PDK1) inhibitor BX912 also prolonged survival of scrapie-infected mice [34]. Protein kinase inhibitors may have good potential in prion disease therapeutics. Unfortunately, the signaling pathways most critical to prion disease pathogenesis have yet to be fully identified.

We have developed a kinomics approach to identify signaling pathways dysregulated during prion disease pathogenesis (Shott et al., companion paper). We initially tested the approach in a simplified in vitro model of prion disease pathogenesis (Shott et al., companion paper). Here, we applied the approach to mice infected with mouse-adapted scrapie. We identified two signaling pathways dysregulated during scrapie pathogenesis. The calcium/calmodulin-dependent protein kinase, beta isoform (CaMK4β)/cAMP response element-binding protein (CREB) signaling pathway, which promotes neuronal survival, was activated at earlier times but its activation state returned to that in mock-infected mice later on. Mammalian STE20-like protein kinase 1 (MST1) signaling, which promotes neuronal death, was, in contrast, activated at these later times. The dysregulation of CaMK4β/CREB and MST1 signaling pathways may therefore be critical to the neurodegeneration in scrapie infected mice.

## Results

### PrP^res^ is first detected in scrapie-infected mice at 130 dpi

Mock-infected mice or mice infected intraperitoneally with scrapie (mouse-adapted strain RML) were euthanized at 70, 90, 110, 130 days post-infection (dpi), or at terminal stages of disease (155–190 dpi). Brains were dissected into *(i)* cortical (cerebrum), *(ii)* subcortical (including thalamus, hypothalamus and hippocampus) and *(iii)* brainstem-cerebellum as described [35].

**Figure 1:**
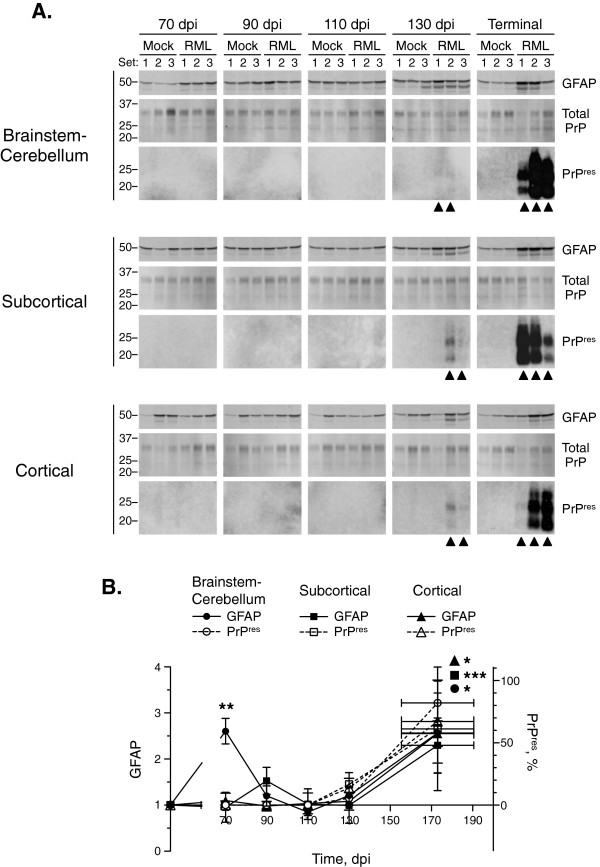
**Levels of GFAP and PrP** ^**res**^ **during scrapie progression. (A)** Western blots of GFAP, total PrP, and PrP^res^ in homogenates of brainstem-cerebellum, subcortical region, and cortical region of three mock (Mock)- or scrapie (RML)-infected mice at 70, 90, 110, 130 dpi or at terminal stage of disease (155–190 dpi). Molecular weights in kDa are indicated on the left. Black triangles, samples in which PrP^res^ was quantitated. **(B)** Time-course changes in the levels of GFAP and PrP^res^. Line graphs show the levels of GFAP in scrapie-infected mice normalized to the average levels in mock-infected mice at each time point. The levels of PrP^res^ are expressed as a percentage relative to the highest level detected in each region. Mean ± SD; *n* = 3 (Mean ± range for PrP^res^ at 130 dpi where *n* = 2). Error bars on the x-axis, range in time of onset of terminal disease. The differences in GFAP levels in scrapie- versus mock-infected mice were analyzed by unpaired two-tail *t*-test. *P < 0.05; **P < 0.01; ***P < 0.001.

We first analyzed the levels of protease-resistant PrP^Sc^ (PrP^res^) and total glial fibrillary acidic protein (GFAP) by Western blot. PrP^res^ was enriched by sodium phosphotungstic acid (NaPTA) precipitation prior to proteinase K (PK) treatment. As expected, PrP^res^ was only detected in scrapie-infected mice, and its levels increased with time of infection (Figure 1A). PrP^res^ was first detected in all regions at 130 dpi, and increased coordinately with the levels of GFAP from 130 dpi to terminal stages of disease (unpaired two-tail *t*-test; GFAP brainstem-cerebellum, *P* = 0.0388; subcortical region, *P* = 0.0008; cortical region, *P* = 0.0414) (Figure 1B), as expected [36, 37]. The levels of GFAP were also higher in the brainstem-cerebellum of scrapie-infected mice prior to PrP^res^ accumulation at 70 dpi (*P* = 0.0041) and showed a tendency to higher levels in the cortical region at 90 dpi, as observed previously [38]. The lower molecular weight form of GFAP has also been observed previously [39] and is likely the result of degradation.

### Primary kinomic screens identified two signaling pathways of potential interest, which are involved in neuronal death and survival

**Figure 2:**
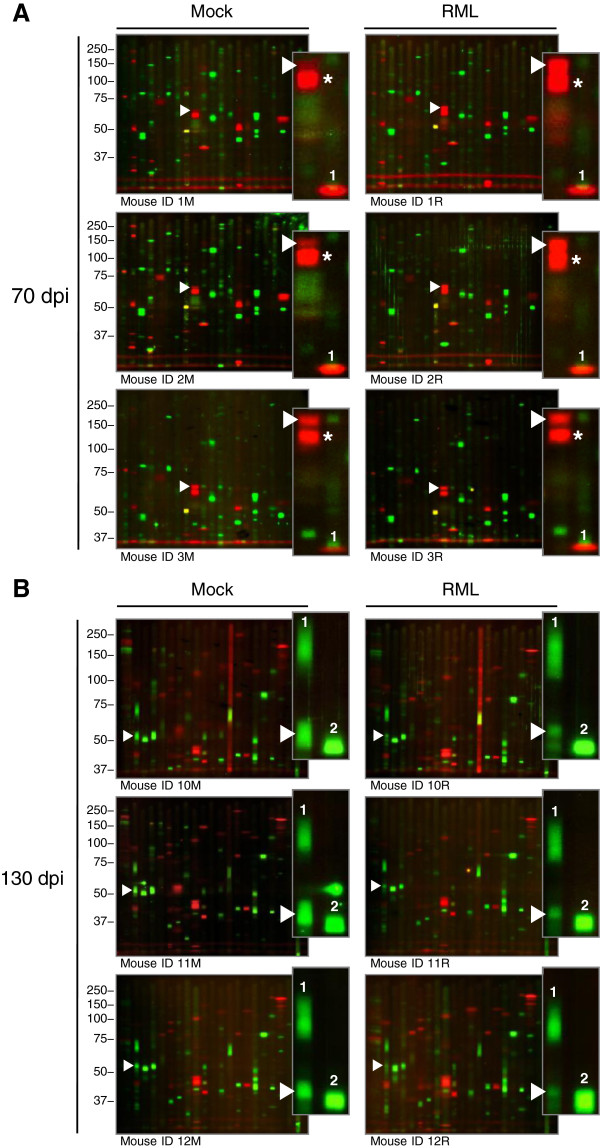
**Differential expression of protein kinases in scrapie- versus mock-infected mice at 70 and 130 dpi.** Primary multiplex Western blots analyzing the expression levels of 139 protein kinases in three mock- or scrapie-infected mice at 70 **(A)** or 130 **(B)** dpi. Mice identified by ID numbers; M, mock-infected; RML, RML-infected. Molecular weights in kDa are indicated on the left. The protein kinases CaMK4β (**A**; probed with antibody set 1) and DLK (**B**; probed with antibody set 2) are indicated by white arrowheads and enlarged to illustrate their differential expression in scrapie- versus mock-infected mice. The antibody used to detect CaMK4β also detects CaMK4 (red band, indicated by the asterisks), the levels of which remain constant. The expression levels of the other protein kinases tested in the same or adjacent lanes also remained constant or were expressed differently than CaMK4β and DLK (**A**, CK1ϵ [red band] indicated by 1; **B**, LIMK1 indicated by 1 and GSK3α indicated by 2). Apparent signal above GSK3α in mouse ID 11 M is an artifact.

After intraperitoneal infection, prion disease spreads in the brain caudal to rostral [40–42]. We therefore selected the brainstem-cerebellum for the primary screens, for the greatest window of opportunity to identify signaling pathways dysregulated during pathogenesis. Primary multiplex Western blots analyzed the expression levels of 139 protein kinases (Additional file 1: Table S1) in brainstem-cerebellum homogenates of three mock-infected and three scrapie-infected mice euthanized at 70, 90, 110, 130 dpi or at terminal stages (155–190 dpi). The antibodies included in our analyses were optimized and validated as described (Shott et al., companion paper). We detected 109 protein kinases (78% of the 139 tested), most of which were differentially expressed in scrapie- as compared to mock-infected mice. For example, CaMK4β was expressed to higher levels in scrapie- than in mock-infected mice at 70 dpi (Figure 2A), and dual leucine zipper kinase (DLK) to lower levels at 130 dpi (Figure 2B). Ten protein kinases were not detected in one set of mock- and scrapie-infected mice at one time point (tropomyosin-related kinase B [TrkB], Set 1 at 70 dpi; membrane-associated tyrosine/threonine-specific cdc2-inhibitory kinase [Myt1], Set 2 at 110 dpi; v-Raf murine sarcoma viral oncogene homolog B1 [B-Raf], ribosomal protein S6 kinase, 70 kilodalton, polypeptide 1 [p70S6K], serine/threonine-protein kinase D3 [PKD3], protein kinase R [PKR], serine/threonine-protein kinase N2 [PRK2], STE20-like serine/threonine-protein kinase [SLK], TRAF2 and NCK-interacting protein kinase [TNIK], and tropomyosin-related kinase C [TrkC], Set 3 at 130 dpi). Five other proteins (calcium/calmodulin-dependent protein kinase 1 alpha [CaMK1α], cyclin D1, cyclin G1, p25 and p35) were not resolved in one set at 70, 90, 110, and 130 dpi. Mitogen-activated protein kinase kinase kinase 5 (ASK1) and mitogen-activated protein kinase kinase kinase 1 (MEKK1) were not quantitated in one set at 90 dpi, or two at terminal stages, due to transfer or blotting artifacts, respectively. The raw densitometric data obtained from all other protein kinases was normalized to data from the mock-infected mice, log_2_ transformed and analyzed by unsupervised hierarchical clustering.

**Figure 3:**
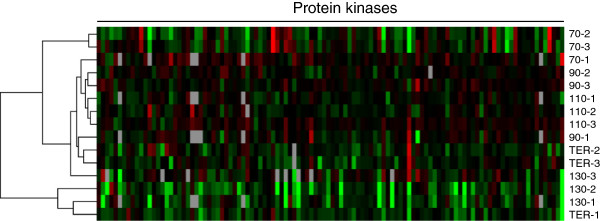
**Blind clustering of scrapie-infected mice results in their grouping largely by time after infection.** Hierarchical clustering of the scrapie-infected mice euthanized at 70, 90, 110, 130 dpi or at terminal stage of disease (TER) using the densitometric data of the 109 protein kinases detected in primary multiplex Western blots. The normalized and log_2_ transformed levels of protein kinase expression for each set (1, 2, 3 - consisting of one scrapie-and one mock-infected mouse euthanized at each time point) were clustered using Gene Cluster 3.0 software (Euclidean distance; complete linkage) and presented using Java Treeview.

We first blindly clustered the scrapie-infected mice to evaluate the changes in protein kinase expression during disease progression (Figure 3). The most distantly related cluster consisted of the two mice euthanized at 130 dpi with the highest levels of PrP^res^, and one mouse euthanized at terminal stages of disease (130–1, 130–2, TER-1). The second most distantly related cluster consisted of two mice euthanized at 70 dpi (70–2, 70–3). All other mice clustered in two subclusters. One of them consisted of the third mouse euthanized at 70 dpi (70–1), two mice euthanized at 90 dpi, and all three mice euthanized at 110 dpi. The other consisted of mice euthanized at 90 dpi, 130 dpi, and terminals stages of disease (90–1, 130–3, TER-2, TER-3). The mice therefore clustered largely based on time after infection, suggesting that levels of protein kinase expression in mice changed in response to scrapie pathogenesis.

**Figure 4:**
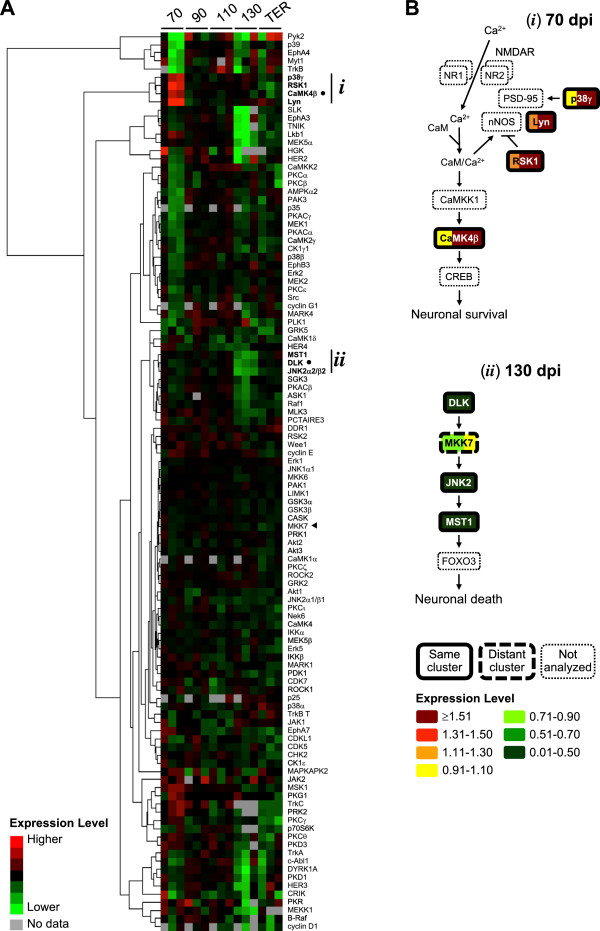
**Identification of two signaling pathways of potential interest involved in neuronal survival and death. (A)** Hierarchical clustering the normalized and log_2_ transformed densitometric data of the expression levels of 109 protein kinases detected in primary multiplex Western blots of brainstem-cerebellums of scrapie-infected mice at 70, 90, 110, 130 dpi or at terminal stage of disease (TER). Red, higher expression level; green, lower expression level; grey, no data (protein kinases that were not resolved, not detected, or not quantitated due to transfer or blotting artifacts). Cluster (*i*) consists of protein kinases involved in the NMDAR-regulated CaMK4β signaling pathway. Cluster (*ii*) consists of protein kinases involved in the MST1 signaling pathway. The protein kinases highlighted in Figure 2, CaMK4β and DLK, are indicated by (●), and MKK7 is indicated by (◄). **(B)** Expression levels of protein kinases involved in the CaMK4β and MST1 signaling pathways, and included in the primary multiplex Western blots, at 70 dpi (top panel) and 130 dpi (bottom panel), respectively. Color bars indicate the levels in each of the three scrapie-infected mice normalized to those in mock-infected mice.

The data was next clustered to identify the expression levels of protein kinases which changed similarly in scrapie-infected mice during disease progression (Figure 4A). Protein kinases with similar changes in expression may, or may not, also be involved in the same signaling pathway. To identify those that were, we performed literature and signal transduction database searches. Mitogen-activated protein kinase 12 (p38γ), ribosomal S6 kinase 1 (RSK1), v-yes-1 Yamaguchi sarcoma viral related oncogene homolog (Lyn), and CaMK4β, which clustered together because they were expressed to similarly higher levels in scrapie- than in mock-infected mice at 70 dpi, are all involved in an *N*-methyl-D-aspartate receptor (NMDAR)-regulated CaMK4β signaling pathway that promotes neuronal survival (Figure 4B, *i*). MST1, DLK and mitogen-activated protein kinase 9 (JNK2) (p54; α2/β2 isoforms), which clustered together because they were expressed to similarly lower levels in scrapie- than in mock-infected mice at 130 dpi, are all involved in an MST1 signaling pathway that promotes neuronal death (Figure 4B, *ii*). Although dual specificity mitogen-activated protein kinase kinase 7 (MKK7), which is also involved in this pathway, clustered distantly, its expression levels were also somewhat lower in scrapie- than in mock-infected mice at 130 dpi.

Primary screens of brainstem-cerebellum homogenates from scrapie-infected mice therefore identified two signaling pathways which may be dysregulated during pathogenesis. The NMDAR-regulated CaMK4β signaling pathway promotes neuronal survival and the expression levels of the protein kinases involved changed (increased) the most at earlier times. Conversely, the MST1 signaling pathway promotes neuronal death, and the expression levels of involved protein kinases were most affected (decreased) at later times.

### CaMK4β/CREB signaling is activated at early stages of scrapie in mice

**Figure 5:**
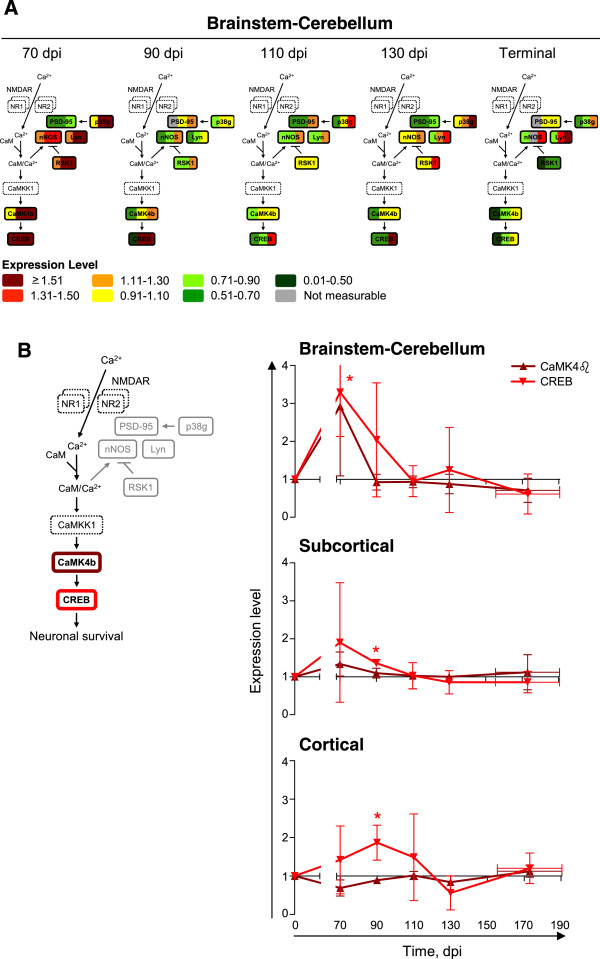
**CREB is expressed to higher levels in scrapie- than in mock-infected mice at 70 and 90 dpi.** Targeted secondary analyses of the NMDAR-regulated CaMK4β signaling pathway in brainstem-cerebellum, subcortical and cortical regions of scrapie-infected mice at 70, 90, 110, 130 dpi or at terminal stage of disease (TER; 155–190 dpi). **(A)** Expression levels of p38γ, Lyn, RSK1, CaMK4β, nNOS, CREB, and PSD-95 in the brainstem-cerebellums of each of the three scrapie-infected mice at each time point, normalized to those in the mock-infected mice, shown by color bars. The proteins in dashed lines were not analyzed. **(B)** Levels of CaMK4β and CREB in all macrodissected regions, normalized to those in the mock-infected mice, shown as time series. Mean ± SD; *n* = 3. Error bars on the x-axes, range in time of onset of terminal disease. The differences in the expression levels in scrapie- versus mock-infected mice were analyzed by two-tailed paired ratio *t*-test. *P < 0.05.

The NMDAR-regulated CaMK4β signaling pathway promotes neuronal survival though the activation of CREB [43]. We therefore analyzed the expression levels of CREB. We also analyzed the expression levels of neuronal nitric oxide synthase (nNOS) and the scaffold protein post-synaptic density protein 95 (PSD-95), which associate with, or are regulated by, RSK1, Lyn and p38γ at NMDARs [44–46]. CaMK4β, CREB, p38γ, RSK1, and PSD-95 levels in the brainstem-cerebellum of scrapie-infected mice were different from those in mock-infected mice (nonlinear regression analysis; CaMK4β, *P* = 0.0404; CREB, *P* = 0.0372; p38γ, *P* = 0.0369; RSK1, *P* = 0.0471; PSD-95, *P* = 0.0147) (Figure 5). Consistent with the higher levels of RSK1, Lyn, p38γ, and CaMK4β in the brainstem-cerebellum of scrapie-infected mice at 70 dpi, the levels of nNOS and CREB were also higher at this time (two-tail paired ratio *t*-test; nNOS, *P* = 0.0434; CREB, *P* = 0.0341) (Figure 5). We expanded our analyses to the subcortical and cortical regions. CREB levels were also significantly higher in the subcortical and cortical regions of scrapie-infected mice, but at 90 dpi (subcortical region, *P* = 0.0196; cortical region, *P* = 0.0481) (Figure 5). CaMK4β and CREB levels changed coordinately in both the brainstem-cerebellum and subcortical regions (Figure 5).

We next performed targeted tertiary (phosphorylation state-specific) analyses to characterize the activation state of (NMDAR-regulated) CaMK4β/CREB signaling in scrapie-infected mice. The activation of Lyn and RSK1 involves autophosphorylation on Y396 or S380, respectively [47–50]. Activated RSK1 (P-S380) inhibits nNOS by phosphorylation on S847 [45]. Activated p38 (P-T180/Y182) phosphorylates PSD-95 on S290 [46]. Activated CaMK4β (P-T196; corresponding to human T200) phosphorylates CREB on S133 [51, 52]. Phosphorylated CREB promotes transcription of genes that encode proteins involved in neuronal survival [53, 54]. There is no antibody specific for T180/Y182 phosphorylation on only p38γ. We therefore used an antibody that detects T180/Y182 phosphorylation on all p38 isoforms (p38α, p38β, p38γ, p38δ). In summary, we analyzed the levels of activating phosphorylation of p38 (P-T180/Y182), Lyn (P-Y396), RSK1 (P-S380), CaMK4β (P-T196), and CREB (P-S133), and the levels of inhibitory phosphorylation of nNOS (P-S847). No antibody specific for p38-phosphorylated PSD-95 (P-S290) was available.

**Figure 6:**
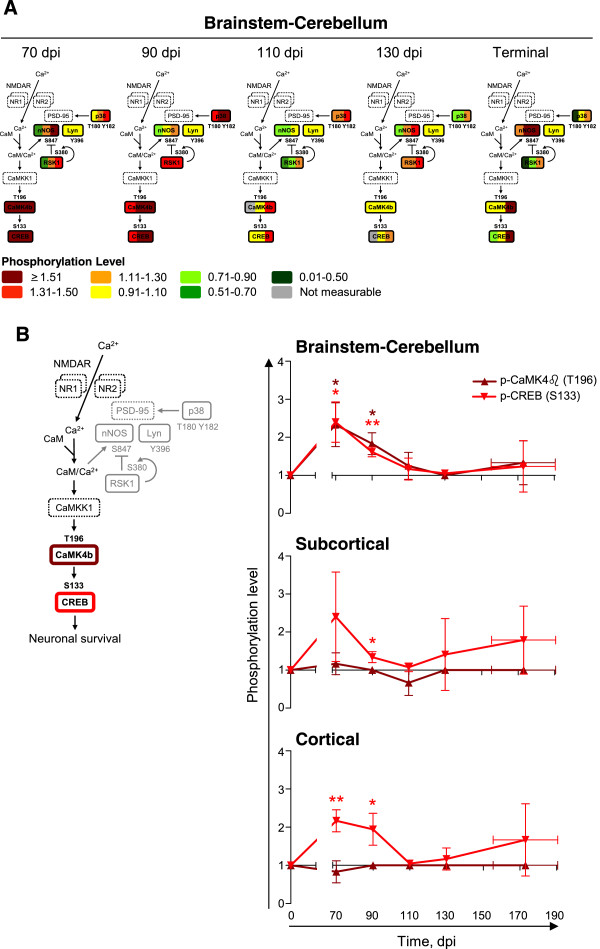
**Activation of CaMK4** **β** **/CREB signaling in scrapie-infected mice early in disease.** Targeted tertiary analyses of the NMDAR-regulated CaMK4β signaling pathway in brainstem-cerebellum, subcortical and cortical regions of scrapie-infected mice at 70, 90, 110, 130 dpi or at terminal stage of disease (TER; 155–190 dpi). **(A)** Levels of phosphorylated p38 (T180/Y182), Lyn (Y396), RSK1 (S380), nNOS (S847), CaMK4β (T196), and CREB (S133) in the brainstem-cerebellums of each of the three scrapie-infected mice at each time, normalized to those in the mock-infected mice, shown by color bars. The proteins in dashed lines were not analyzed. **(B)** Levels of phosphorylated CaMK4β (T196) and CREB (S133) in all macrodissected regions, normalized to those in the mock-infected mice, shown as time series. Mean ± SD; *n* = 3 (Mean ± range for CaMK4β (T196) at 90 dpi [subcortical region] and 110 dpi [brainstem-cerebellum], and CREB (S133) at 130 dpi [brainstem-cerebellum]; *n* = 2). Error bars on the x-axes, range in time of onset of terminal disease. The differences in phosphorylation levels in scrapie- versus mock-infected mice were analyzed by two-tailed paired ratio *t*-test. *P < 0.05; **P < 0.01.

As their total levels, the levels of activated CaMK4β (P-T196) and CREB (P-S133) changed coordinately. Their levels were significantly higher in the brainstem-cerebellum of scrapie-infected than of mock-infected mice at 70 and 90 dpi (two-tail paired ratio *t*-test; CaMK4β *P* = 0.0256 [70 dpi], 0.0248 [90 dpi]; CREB *P* = 0.0197 [70 dpi], 0.0086 [90 dpi]) (Figure 6). The levels of phosphorylated CREB in the subcortical and cortical regions were also significantly higher (subcortical region *P* = 0.0412 [90 dpi]; cortical region *P* = 0.0093 [70 dpi], 0.0376 [90 dpi]), or at least there was a trend to higher levels in scrapie-infected than in mock-infected mice (subcortical region *P* = 0.0936 [70 dpi]) (Figure 6). The levels of activated CaMK4β in the subcortical and cortical regions at 70 and 90 dpi were similar in scrapie- and mock-infected mice. The levels of activated p38 (P-T180/T182) were significantly higher in scrapie-infected than in mock-infected mice at 70 and 90 dpi (brainstem-cerebellum *P* = 0.0225 [90 dpi]; subcortical region *P* = 0.0106 [70 dpi]; cortical region *P* = 0.0014 [70 dpi]) but also at 110 and 130 dpi (brainstem-cerebellum *P* = 0.0465 [110 dpi]; cortical region *P* = 0.0116 [130 dpi]) (Figure 6). There was no correlation between the levels of activated RSK1 (P-S380) and nNOS (P-S847) in the different brain regions (Additional file 2: Figure S1). The levels activated Lyn (P-Y396) were mostly unchanged in scrapie-infected mice relative to mock-infected mice.

In summary, CaMK4β/CREB signaling was activated in scrapie-infected mice at early times. The expression levels and the levels of phosphorylated CREB (P-S133) and activated CaMK4β (P-T196) were higher in scrapie- than in mock-infected mice at 70 and 90 dpi.

### MST1 is activated at late stages of mouse scrapie

**Figure 7:**
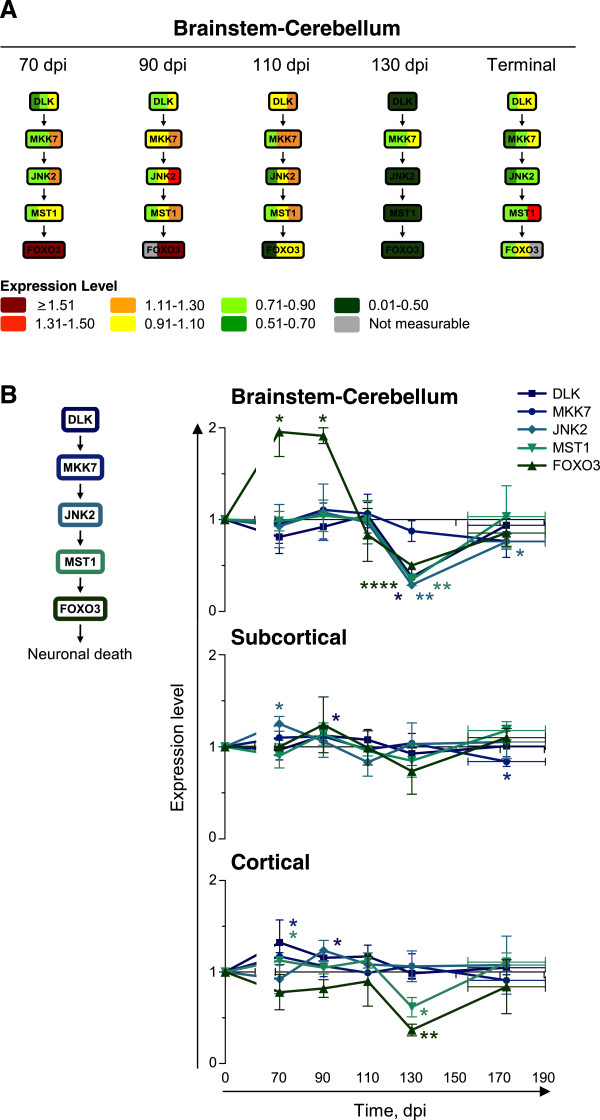
**MST1 and FOXO3 are expressed to lower levels in scrapie-infected mice at 130 dpi.** Targeted secondary analyses of the MST1 signaling pathway in brainstem-cerebellum, subcortical and cortical regions of scrapie-infected mice at 70, 90, 110, 130 dpi or at terminal stage of disease (TER; 155–190 dpi). **(A)** Levels of DLK, MKK7, JNK2, MST1, and FOXO3 in the brainstem-cerebellums of three scrapie-infected mice at each time point, normalized to those in the mock-infected mice, shown by color bars. **(B)** Protein levels in all macrodissected regions, normalized to those in mock-infected mice, shown as time series. Mean ± SD; *n* = 3 (Mean ± range for FOXO3 at 90 dpi [brainstem-cerebellum] and TER [brainstem-cerebellum, subcortical region]; *n* = 2). Error bars on the x-axes, range in time of onset of terminal disease. The differences in expression levels in scrapie- versus mock-infected mice were analyzed by two-tailed paired ratio *t*-test. *P < 0.05; **P < 0.01; ****P < 0.0001.

The MST1 signaling pathway mediates neuronal death by activating forkhead box protein O3 (FOXO3) [55]. We therefore analyzed the expression levels of FOXO3 in targeted secondary Western blots of brainstem-cerebellum homogenates from scrapie-infected mice at 70, 90, 110, 130 dpi or at terminal stages of disease. Consistent with the lower expression levels of DLK (two-tailed paired ratio *t*-test; *P* = 0.0182), JNK2 (*P* = 0.0063), and MST1 (*P* = 0.0095), FOXO3 levels were also significantly lower (*P* < 0.0001) in the brainstem-cerebellum of scrapie- than of mock-infected mice at 130 dpi (Figure 7). The levels of DLK, MKK7, and JNK2 were more similar in the subcortical and cortical regions of mock- and scrapie-infected mice than in their brainstem-cerebellum (Figure 7) (Additional file 3: Figure S2). However, MST1 and FOXO3 were still expressed to significantly lower levels in the cortical region of scrapie- than of mock-infected mice at 130 dpi (MST1, *P* = 0.0384; FOXO3, *P* = 0.0090) (Figure 7). MST1 levels also appeared to be different in the subcortical region of scrapie- or mock-infected mice (nonlinear regression analysis; MST1, *P* = 0.0513), but did not reach statistical significance at any single time point. The differential expression of MST1 and FOXO3 in the brainstem-cerebellum and cortical region, albeit to differing degrees, supports a model in which MST1/FOXO3 signaling is dysregulated during progression of scrapie.

To test this model, we performed targeted (phosphorylation state-specific) tertiary Western blots to characterize the activation state of MST1/FOXO3 signaling. MST1 is activated by autophosphorylation on T183 (P-T183) [56]. Activated MST1 phosphorylates FOXO3 in the cytosol on S208 (corresponding to S207 in human) [55]. Phosphorylated FOXO3 (P-S208) then translocates to the nucleus where it activates transcription of genes that encode proteins involved in neuronal death. Activated MST1 is further activated by caspase-3 cleavage. Cleaved MST1 further promotes cell death [57–60]. We analyzed the levels of activating phosphorylation of MST1 (P-T183) and FOXO3 (P-S207) and activating cleavage of MST1. MST1 activation is promoted by JNK-mediated phosphorylation on S82 [61] and active JNK2 is phosphorylated on T183/Y185. As no antibody available was specific for P-T183/Y185 of only JNK2 or P-S82 of MST1, we used antibodies that detect T183/Y185 phosphorylation on all JNK isoforms (JNK1, JNK2, JNK3; p46 [JNK1α1, JNK1β1, JNK2α1, JNK2β1, JNK3α1] and p54 [JNK1α2, JNK1β2, JNK2α2, JNK2β2, JNK3β2]) or autophosphorylated MST1 (P-T183).

**Figure 8:**
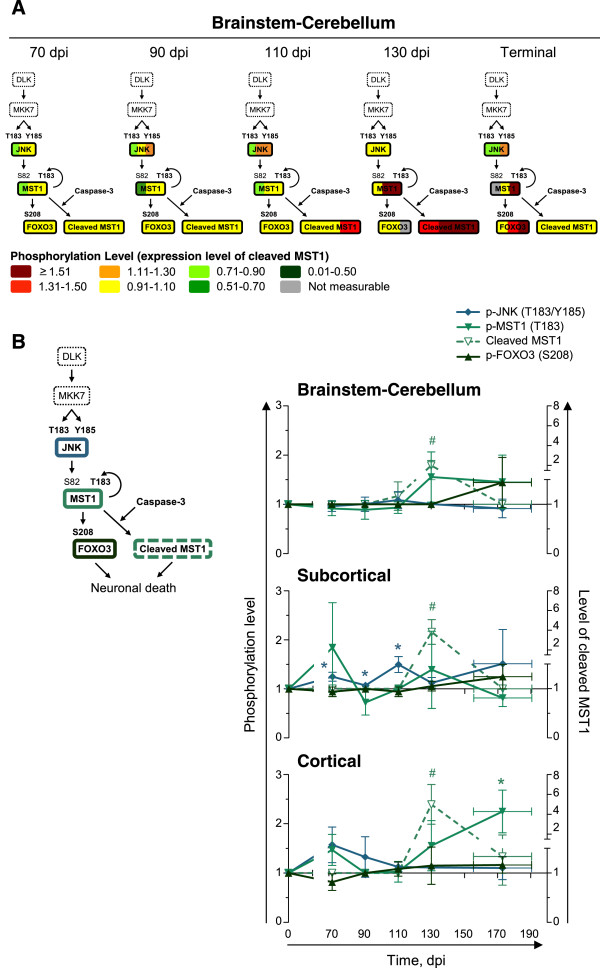
**Activation of MST1 in scrapie-infected mice at late stages of disease.** Targeted tertiary analyses of MST1 signaling in brainstem-cerebellum, subcortical region, and cortical region of scrapie-infected mice at 70, 90, 110, 130 dpi or at terminal stage of disease (TER; 155–190 dpi). **(A)** Levels of phosphorylated JNK (T183/Y185), MST1 (T183), FOXO3 (S208), and cleaved MST1, in the brainstem-cerebellums of each of the three scrapie-infected mice at each time point, normalized to those in the mock-infected mice, shown by color bars. The proteins in dashed lines were not analyzed. **(B)** Phosphorylation levels in all brains regions, normalized to those in the mock-infected mice, shown as time series. Mean ± SD; *n* = 3 (Mean ± range for MST1 (T183) at 110 dpi [subcortical region] and TER [brainstem-cerebellum], and FOXO3 (S208) at 130 dpi [brainstem-cerebellum] and TER [cortical region]; *n* = 2). Error bars on the x-axes, range in time of onset of terminal disease. The differences in the phosphorylation levels, or levels of cleaved MST1, in scrapie- versus mock-infected mice were analyzed by two-tailed paired ratio *t*-test. *(^#^, cleaved MST1), *P* < 0.05.

There was a trend to higher levels of activated JNK (p54; P-T183/Y185) and MST1 (P-T183) in the cortical region of scrapie-infected mice at 70 dpi (two-tail paired ratio *t*-test; JNK [p54], *P* = 0.0851; MST1, *P* = 0.0880) (Figure 8). The levels of activated JNK were significantly higher in the subcortical region at 70 dpi (*P* = 0.0332), whereas those of MST1 were not (*P* = 0.2222). Cleaved MST1 and phosphorylated FOXO3 (P-S208) levels were similar in scrapie- and mock-infected mice at this time (Additional file 3: Figure S2). Levels of cleaved MST1 were significantly higher at 130 dpi in all brain regions (brainstem-cerebellum, *P* = 0.0452; subcortical region, *P* = 0.0237; cortical region, *P* = 0.0243), whereas activated JNK levels were similar in scrapie- and mock-infected mice at this time (Figure 8). Levels of activated MST1 (P-T183) were significantly higher (*P* = 0.0215) in the cortical region of scrapie-infected mice at terminal stages, when the levels of cleaved MST1 had returned to levels similar to those in mock-infected mice.

In summary, MST1 signaling was activated in scrapie-infected mice at late times. Levels of cleaved MST1 were higher at 130 dpi, when total levels of MST1 and FOXO3 were lower. At terminal stages of disease, levels of activated MST1 (P-T183) were higher in scrapie- than in mock-infected mice.

## Discussion

**Figure 9:**
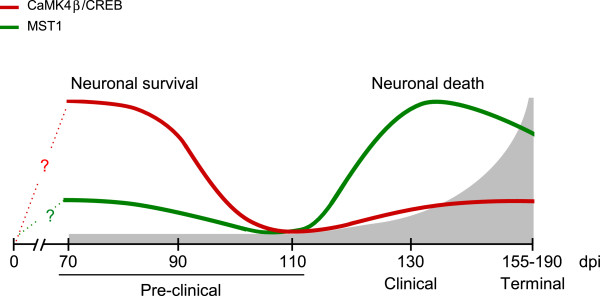
**A model for the activation of signaling pathways involved in neuronal survival and death during scrapie pathogenesis.** Relative activation states of CaMK4β/CREB (red line) and MST1 (green line) signaling in scrapie-infected mice during disease progression, indicated in grey. Between 90 and 130 dpi, there is a switch from signaling involved in neuronal survival (activated CaMK4β/CREB) to that involved in neuronal death (activated MST1). Adapted from [72] (Figure 7).

We describe the application of a new kinomics approach to an in vivo model of prion disease pathogenesis, mice intraperitoneally infected with scrapie strain RML. The primary screens identified CaMK4β and MST1 signaling pathways as of potential interest. Targeted analyses then tested the activation state of these pathways. CaMK4β/CREB signaling, which promotes neuronal survival, was activated at earlier times in scrapie-infected mice, but returned to the levels of mock-infected mice at later times. At these later times, MST1 signaling, which promote neuronal death, was activated (Figure 9).

The activation of CaMK4β/CREB signaling at pre-clinical stages of prion disease had not been described. CREB is critical to neuronal survival. CREB/cAMP response element modulator (CREM) double knockout mice, or mice in which CREB is inhibited by overexpression of a dominant negative mutant or inhibitory peptides, suffer extensive neuronal loss [62–64]. Active CREB promotes neuronal survival by regulating transcription of “activity-regulated inhibitor of death” (AID) genes, including *gadd45*β, *gadd45*γ, *btg2*, *npas4*, *nr4a1*, *inhba*, *atf3*, *ifi202b*, *serpinb2* [53, 54]. CREB also regulates the transcription of miR132-3p [65, 66], which modulates synapse morphology [67]. High-throughput analyses have identified changes in many mRNAs and miRNAs, including miR132-3p, in scrapie-infected mice prior to the accumulation of PrP^res^ or the onset of clinical disease [68–73]. Elevated levels of miR132-3p and AID genes (*gadd45*β, *gadd45*γ, *btg2*, *npas4*, *nr4a1*) were observed at 70–110 dpi after infection with the same scrapie strain and by the same route of inoculation as in the current experiments [72]. Activated CaMK4β/CREB signaling may well promote neuronal survival early in prion infection by upregulating the expression of miR132-3p and AID genes.

CaMK4β/CREB signaling returned to the activation levels of mock-infected mice at 110 dpi. Calcineurin regulates the activity of CaMK4 [74] and CREB [75], and elevated calcineurin activity was observed in scrapie-infected mice at clinical stages of disease [19]. Moreover, the calcineurin inhibitor FK506 prolonged survival of scrapie-infected mice and inhibited neuronal death in cultured neurons treated with PrP106-126 [19, 76, 77]. CaMK4β/CREB signaling is therefore most likely neuroprotective in scrapie-infected mice. Decreased levels of activated CaMK4β (P-T196) and phosphorylated CREB (P-S133) were associated with decreased expression levels, suggesting that degradation may be involved in downregulation of this pathway. CaMK4 and CREB are degraded by calpain, a calcium-dependent protease [78, 79]. Active calpain degraded CaMK4 and CREB in cultured neurons treated with hydrogen peroxide after CREB (P-S133) dephosphorylation [80]. Increased levels of calpain have been observed in in vitro and in vivo models of prion disease [81, 82], and calpain inhibition limited neuronal death in prion-infected cultured organotypic cerebellar slices or cultured neurons treated with PrP106-126 [81, 83, 84]. CaMK4β/CREB signaling activation may be inhibited in scrapie-infected mice after 90 days by dephosphorylation and degradation mediated by calpain. CaMK4 and CREB are also dephosphorylated by protein phosphatase 2A (PP2A) [85–87], the activity of which has yet to be evaluated during scrapie infection.

MST1 signaling was activated at 130 dpi, which had not been described in prion disease. MST1 has been shown to be involved in other neurodegenerative diseases. Genetically modified mice that model amyotrophic lateral sclerosis (ALS) lose fewer neurons and survive longer if they are knocked out of MST1 [88]. Active MST1 (P-T183) is cleaved by active (cleaved) caspase-3 [57–60], the levels of which are elevated in scrapie-infected mice (before the accumulation of PrP^res^) and in cultured neurons or neuroblastoma cells exposed to PrP106-126 or PrP^Sc^ [89–92]. We observed elevated levels of cleaved MST1 at 130 dpi, suggesting that it is also cleaved by caspase-3 in vivo. Although cleaved MST1 retains the T183 phosphorylation site, T183 phosphorylation is not required for cleavage [56, 93], and we did not detect cleaved MST1 with the phosphorylation-specific antibody. Caspase-cleaved MST1 translocates to the nucleus and induces chromatin condensation by inducing phosphorylation of histone H2B on S14 [94]. Chromatin condensation has been observed previously in scrapie-infected GT1 cells and mice [95–98]. However, caspase-3 activation is not required for MST1 activation [56] or neuronal death in prion disease [92, 99, 100].

MST1 activates FOXO3, which is otherwise maintained in the cytosol in an inactive state by interaction with 14-3-3 proteins [101]. Cleaved MST1 predominately localizes to the nucleus and is therefore unable to phosphorylate FOXO3 [102, 103]. The levels of phosphorylated FOXO3 (P-S208) might have been higher (but were not statistically different) after the levels of cleaved MST1 had returned to those in mock-infected mice. Active FOXO3 upregulates transcription of genes encoding pro-apoptotic proteins including Bim (bcl-2 interacting mediator of cell death; *bcl2l11*), Puma (Bcl-2-binding component 3; *bbc3*) and Noxa (phorbol-12-myristate-13-acetate-induced protein 1; *pmaip1*) [104–106]. The levels of Bim, Puma, *bcl2l11*, *bbc3* and *pmaip1* are elevated in scrapie-infected mice at late stages of disease progression [72, 107, 108], suggesting that neuronal death mediated by MST1 signaling could involve FOXO3.

The CaMK4β/CREB and MST1 signaling pathways were identified in our screens because the expression levels of the involved protein kinases changed coordinately during prion disease progression (Figures 5 and 7). The CaMK4β/CREB signaling pathway promotes neuronal survival. CaMK4β and CREB were expressed to higher levels and activated at earlier stages of disease. Neurons may activate this pathway to protect themselves from prion-mediated death. Later (at 110 dpi), the neuroprotective CaMK4β/CREB signaling was lost and MST1 signaling was activated. Activation of MST1 signaling was associated with lower levels of full length MST1 and FOXO3. The decrease in full length MST1 was two-fold greater than the increase in the cleaved form (detected in the same blots with the same antibody). Cleaved MST1 may be less stable than full length MST, or full length MST1 may be processed by caspase-dependent and independent pathways.

The opposing changes in expression and activation state suggest an attempt to prevent the neuronal death that would result from activated MST1 signaling. The levels of upstream kinases DLK, MKK7, and JNK2 were also lower in scrapie-infected mice at 130 dpi, albeit only in the brainstem-cerebellum (Figure 7). The cerebellum (in the brainstem-cerebellum) may contain ~50% of all neurons in an adult mouse brain [109] and loses the most neurons in RML-infected mice, as indicated by nuclear DNA fragmentation [96]. The changes in the total levels of proteins involved in the MST1 signaling pathway in each brain region may reflect the differences in the number of affected neurons in each brain region.

The different numbers of affected neurons in each region may also be reflected in the higher levels of total and phosphorylated CaMK4β in the brainstem-cerebellum than in the subcortical or cortical regions. Regardless of absolute numbers, higher levels of total and phosphorylated CREB were expressed in all brain regions. Although CaMK4 (P-T196) could also be responsible for CREB (P-S133) phosphorylation, there were no differences in the levels of activated CaMK4 (P-T196) in scrapie- versus mock-infected mice (data not shown). CREB is also phosphorylated on S133 by other protein kinases, including RSK, cAMP-dependent protein kinase (PKA), and mitogen-activated protein kinase-activated protein kinase 2 (MAPKAPK2) (for a review, see [110]). There was no correlation between the levels of active RSK1 (P-S380) and phosphorylated CREB (P-S133). We did not evaluate other protein kinases upstream of CREB because they were not identified in the primary screening. However, CREB may well be activated independently of CaMK4β in the cortical region or subcortical region of scrapie-infected mice.

The data presented cannot discriminate whether the observed changes in signaling pathways are a cause or a consequence of the pathology. Neither can discriminate whether they result from a loss of PrP^C^ function, a gain of PrP^res^ function, or an altered signaling function resulting from the progressive PrP^C^ misfolding into PrP^res^. Moreover, the dysregulation could have been directly triggered by PrP^res^ or PrP^C^ acting on the neurons, or mediated by the glial alterations that occur during disease progression. The data presented does not resolve either whether the observed signaling changes are specific for prion diseases, or common to other neurodegenerative diseases. Future work will have to address these possibilities.

## Conclusion

We used a kinomics approach to identify two dysregulated signaling pathways, involved in neuronal survival/death, in scrapie-infected mice. Their dysregulation at different times during disease is temporally consistent with the neuronal loss during prion disease. Our findings identified novel signaling involved in prion-mediated neuronal death in vivo and identify potential targets for intervention. It is possible to test now the roles of these signaling pathways in prion disease, as well-characterized inhibitors of several of these proteins are available.

## Materials and methods

### Ethics statement

All of the procedures involving live animals were approved by the Canadian Science Centre for Human and Animal Health – Animal Care Committee (CSCHAH-ACC) or the University of British Columbia Animal Care Committee according to the guidelines set by the Canadian Council on Animal Care. The approval identifications for this study were animal use document (AUD)#H-08-009 and AUD#H-11-020.

### Animals and sample collection

CD1 mice (Charles River Laboratories) between 4–6 weeks of age were infected intraperitoneally with the Rocky Mountain Laboratory (RML) mouse-adapted strain of scrapie. The inoculums consisted of 200 μl of 1% brain homogenate in PBS from either clinically ill or normal control mice. Animals were sacrificed at 70, 90, 110, 130 days post infection (dpi) and terminal disease (155–190 dpi). Clinical signs depicting terminal disease consisted of kyphosis, dull ruffled coat, weight loss of 20% or more and ataxia. Brain samples were collected and macrodissected into three sections, *(i)* cortical, *(ii)* subcortical (including thalamus, hypothalamus and hippocampus) and *(iii)* brainstem-cerebellum. Each section was flash frozen using a dry ice/methanol mixture and stored at −80°C until processing. A total of 3 scrapie- and 3 mock-infected samples were collected per time point.

### Brain homogenization

All procedures were performed at 4°C or on ice. Weighed frozen brainstem-cerebellum, subcortical, and cortical regions from mock- or scrapie-infected mice were homogenized in 3 mL of freshly prepared lysis buffer (20 mM MOPS [pH 7.0], 2 mM EGTA, 5 mM EDTA, 1% Nonidet P-40, 0.01% phosphatase inhibitor cocktail [Pierce, Rockford, Illinois, USA], 0.02% protease inhibitor cocktail [Sigma-Aldrich, St. Louis, Missouri, USA], 10 mM DTT, pH 7.2) [111] per 250 mg of brain, using a tissue homogenizer with disposable tips (TH and hard tissue OMNI tip, respectively; OMNI International, Kennesaw, Georgia, USA). Brain homogenates were passed twice through a 21 gauge needle, sonicated five times for 20 s intervals at 88 W output (431C1 cup horn probe, S-4000 sonicator; Qsonica, Newtown, Connecticut, USA), and pre-cleared by centrifugation for 30 min at 14,000 × *g* (JLA 16.250 BC rotor, Avanti J-E centrifuge; Beckman/Coulter, Brea, California, USA). Approximately 200 μL aliquots of supernatant were aliquoted into 1.5 mL tubes, snap frozen in liquid nitrogen and stored at −80°C.

### Protein quantitation

Protein concentration was determined by Bradford’s assay (Bio-Rad Laboratories, Hercules, California, USA). Protein concentration and equal sample loading was re-tested in preliminary sodium dodecyl sulfate polyacrylamide gel electrophoresis (SDS-PAGE). Brain homogenates were mixed with an equal volume of 2X SDS loading buffer (125 mM Tris-Cl [pH 6.8], 20% glycerol, 4% SDS, 0.005% bromophenol blue, 260 mM DTT) and denatured by incubation at 100°C for 10 min. Afterward, 15-well 8% SDS-PAGE gels (Mini-PROTEAN; Bio-Rad Laboratories) were loaded with 40 μg of denatured protein per linear cm of well (running buffer; 190 mM glycine, 24.8 mM Tris, 0.1% SDS, pH 8.3). Proteins were run through the stacking gel at 50 V, and then resolved for 90 min at 100 V, always at room temperature. Proteins were stained with Coomassie blue G-250 (Bio-Safe Coomassie; Bio-Rad Laboratories) according to the manufacturer’s instructions. Signal from Coomassie-stained protein was detected using an Odyssey infrared imaging system (LI-COR Biosciences, Lincoln, Nebraska, USA) in the 700 nm channel and quantitated using Odyssey 3.0 software (LI-COR Biosciences). Protein amounts were calculated relative to a pre-quantitated standard brain homogenate.

### Sodium phosphotungstic acid precipitation

Sodium phosphotungstic acid (NaPTA) precipitation was adapted from [112, 113]. One milligram of mouse brain homogenate was mixed with an equal volume of 4% sodium lauroylsarcosine (sarkosyl) in PBS. Samples were incubated for 10 min at 37°C with constant agitation (1000 rpm, Thermomixer; Eppendorf, Hamburg, Germany). A 37°C NaPTA solution (PBS, 4% NaPTA, 170 mM MgCl_2_, pH 7.4) was then added to a final concentration of 0.3% NaPTA. Samples were incubated for 60 min at 37°C with constant agitation (1000 rpm, Thermomixer; Eppendorf), then centrifuged at 37°C for 30 min at 16,000 × *g* (FA-45-18-11 rotor, 5418 microfuge; Eppendorf). Pellets were resuspended in 5 μL of 0.1% sarkosyl in PBS and digested with 20 μg of proteinase K (PK; in 0.01 M CaCl_2_) (Roche, Indianapolis, Indiana, USA) for 30 min at 37°C with a brief vortex after 15 min [114]. Digestion was stopped and PK-resistant protein was denatured by quickly adding an approximately equal volume 5X SDS loading buffer (300 mM Tris-Cl [pH 6.8], 50% glycerol, 25% β-mercaptoethanol, 10% SDS, 1% bromophenol blue), and immediately incubating at 100°C for 10 min. Samples were resolved by SDS-PAGE and analyzed by Western blot.

### Western blot

All procedures were performed at room temperature and all washes were performed using gentle rocking, unless otherwise indicated.

PrP^res^ was analyzed in 1.0 mg of NaPTA-enriched, PK-treated mouse brain homogenate using 15-well 12% SDS-PAGE gels (NuPAGE Novex Bis-Tris; Life Technologies Inc., Carlsbad, California, USA). The running buffer (50 mM MOPS, 50 mM Tris, 1 mM EDTA, 0.1% SDS, pH 7.7) within the upper (cathode) chamber contained 5 mM sodium bisulfite. Proteins were run through the stacking gel at 60 V, and then resolved for 2.5 h at 120 V. Afterward, polyvinylidene fluoride (PVDF) membranes (Immuno-Blot, 0.2 μm; Bio-Rad Laboratories) were soaked in methanol for 2 min, then equilibrated in transfer buffer (190 mM glycine, 24.5 mM Tris, 10% methanol) for 20 min. Filter paper (2 sheets/membrane) were equilibrated in transfer buffer for 5 min. Proteins were transferred at 4°C for 2 h at 30 V. After transfer, membranes were dried, soaked in methanol for 2 min and washed twice for 10 min each in Tris-buffered saline (TBS; 140 mM NaCl, 3 mM KCl, 25 mM Tris, pH 7.6). Membranes were blocked for 1 h in TBST (TBS/0.1% Tween-20) with 5% milk, then probed with PrP primary antibody (clone SAF83; a kind gift from Dr. Deborah McKenzie, University of Alberta) diluted to 1:10,000 in TBST with 5% milk for 18 h at 4°C. Afterward, membranes were washed with TBST once for 5 min and thrice for 10 min each. Membranes were incubated with goat anti-mouse horseradish peroxidase (HRP)-labeled secondary antibody (Bio-Rad Laboratories) diluted to 1:40,000 in TBST with 5% milk for 1 h. Membranes were washed in TBST once for 5 min and thrice for 15 min each, incubated for 5 min with enhanced chemiluminescent substrate (SuperSignal West Femto; Pierce) and then exposed to film (Super RX; Fujifilm, Tokyo, Japan). Exposed film was developed and scanned (CanoScan LiDE 200; Canon, Tokyo, Japan). Signal was quantitated using ImageJ (Version 1.47c; National Institutes of Health, Bethesda, Maryland, USA).

To analyze GFAP and total PrP, brain homogenates were mixed with a one-fourth volume of 5X SDS loading buffer. Then, 100 μg of denatured protein was loaded per linear cm onto 15-well 12% SDS-PAGE gels (Mini-PROTEAN; Bio-Rad Laboratories). Proteins were run through the stacking gel at 50 V, and then resolved for 100 min at 100 V. Afterward, gels were equilibrated in transfer buffer (384 mM glycine, 49.6 mM Tris, 20% methanol, 0.01% SDS) [115] for 30 min. PVDF membranes and filter paper (2 sheets/membrane) were also equilibrated in transfer buffer for 20 and 5 min, respectively. Proteins were transferred for 23 h at 4°C; 1 h at 54 mA, 4 h at 189 mA, 8 h at 270 mA and 10 h at 378 mA. Membranes were blocked with 10% blocking buffer (Sigma-Aldrich) for 1 h and probed simultaneously with primary antibodies specific for PrP (clone SAF83) and GFAP (Abcam Inc., Cambridge, Massachusetts, USA) diluted to 1:10,000 and 1:20,000 in 10% blocking buffer with 0.1% Tween-20, respectively, for 18 h at 4°C. Afterward, membranes were washed with TBST once for 5 min and thrice for 10 min each. Membranes were then incubated for 1 h with donkey anti-mouse IRDye 680- (LI-COR Biosciences) and donkey anti-rabbit IRDye 800- (LI-COR Biosciences) labeled secondary antibodies diluted to 1:20,000 in 10% blocking buffer with 0.1% Tween-20 and 0.01% SDS. Afterward, membranes were washed in TBST thrice for 10 min each, and once with TBS for 5 min. Signal from pre-stained protein standards and IRDye 680-labeled secondary antibody was detected at 700 nm using an Odyssey infrared imaging system (LI-COR Biosciences). Signal from IRDye 800-labeled secondary antibody was detected at 800 nm. Signal was quantitated using Odyssey 3.0 software (LI-COR Biosciences). Membranes were then stained with Coomassie blue R-250 (Bio-Rad Laboratories) for 10 min before destaining with 40% methanol in 10% glacial acetic acid thrice for 10 min each, or until excess stain was removed. Signal from Coomassie-stained protein was detected at 700 nm using the Odyssey and quantitated using Odyssey 3.0 software.

For multiplex Western blots, brain homogenates were mixed with an equal volume of 2X, or one-fifth volume of 6X (375 mM Tris-Cl [pH 6.8], 60% glycerol, 12% SDS, 0.015% bromophenol blue, 780 mM DTT), SDS-PAGE loading buffer, and then 200 μg of denatured protein was loaded per linear cm of single-well 8% SDS-PAGE gels (Mini-PROTEAN; Bio-Rad Laboratories). Proteins were resolved as described for protein quantitation and transferred as described for Western blots of GFAP and total PrP. Dried membranes were probed immediately or stored at −30°C. All multiplex Western blots were performed in three sets, each composed of one membrane from a mock- and one from a scrapie-infected mouse euthanized at 70, 90, 110, 130 dpi, or at terminal stage of disease. Membranes were blocked for 1 h with 10% blocking buffer (Sigma-Aldrich) for evaluation of total protein levels, or in 3% BSA (Rockland, Gilbertsville, Pennsylvania, USA) for evaluation of phosphorylation levels. Membranes were rinsed briefly with TBS and positioned within a 24-lane multi-screen apparatus (MPX; LI-COR Biosciences). Combinations of primary antibodies were diluted in 10% blocking buffer or 3% BSA, as appropriate, with 0.1% Tween-20. One hundred sixty microliters of each antibody dilution was loaded in each lane of the multi-screen apparatus. After incubation for 18 h at 4°C, membranes were briefly washed once with TBST within the multi-screen apparatus then removed from the apparatus and further washed in TBST, once for 5 min and four times for 15 min each. Membranes were incubated with secondary antibody diluted to 1:20,000 in 10% blocking buffer or 3% BSA, as appropriate, with 0.1% Tween-20 and 0.01% SDS for 1 h. Mouse monoclonal primary antibodies were detected with donkey anti-mouse IRDye 680-labeled secondary antibody. Rabbit or goat primary polyclonal primary antibodies were detected with donkey anti-rabbit or donkey anti-goat IRDye 800-labeled secondary antibody (LI-COR Biosciences), respectively. Membranes were washed in TBST four times for 15 min each and once with TBS for 5 min. Signal from IRDye 680- and IRDye 800-labeled secondary antibody was detected at 700 and 800 nm, respectively, using the Odyssey system. Signal was quantitated using Odyssey 3.0 software.

Membranes from scrapie-infected mice were stripped (only once) together and in parallel with the membranes from the mock-infected mice from the same set, under conditions to minimize protein loss [116]. Membranes were stripped with mild stripping buffer (25 mM glycine, 1% SDS, pH 2.0) once for 5 min and four times for 15 min each, then with harsh stripping buffer (50 mM Tris-Cl [pH 7.0], 2% SDS, 50 mM DTT) [117] once for 15 min at 37°C. Afterward, stripped membranes were washed with TBST once and TBS once for 5 min each, then blocked and reprobed with a second set of primary antibodies as described.

### Hierarchical cluster analysis

For each set, the densitometric data from the primary multiplex Western blots of brainstem-cerebellum homogenates of scrapie-infected mice was normalized to that from the mock-infected mice. Relative protein kinase expression levels were then log_2_ transformed and analyzed with Gene Cluster 3.0 [118] using Euclidean distance and complete linkage. Java Treeview was used to present the clusters [119].

### Statistical analyses

All data was analyzed using Prism (Version 5.0f; GraphPad Software Inc., La Jolla, California, USA). The targeted Western blots were performed in three experimental sets on different days. Each set encompassed one scrapie- and one mock-infected mouse from each time point. The ratios from each set were thus analyzed by paired *t*-test. The ratios were log transformed before analyses to transform the increased and decreased ratios (ratios greater or smaller than 1, respectively) to proportional values. For nonlinear regression analyses, curves of the normalized total and phosphorylation protein levels in scrapie-infected mice were compared to no changes (a line with intercept = 1, slope = 0), representing the levels in mock-infected mice, using a replicates test for lack-of-fit.

## Electronic supplementary material

Additional file 1: Table S1: Accession numbers for the 130 protein kinases and 9 regulatory subunits analyzed in the primary multiplex Western blots. One hundred and twenty-four protein kinases or regulatory subunits included in our multiplex Western blots were detected in 200 μg of mouse brain homogenate per linear well cm. The other 15 (indicated by the asterisks) were detected in multiplex Western blots using an equivalent amount of lysate from cycling 3T3 mouse fibroblasts. The human accession number for each protein is indicated. (XLS 42 KB)

Additional file 2: Figure S1: CREB is expressed and phosphorylated to higher levels in the subcortical and cortical regions of scrapie- than mock-infected mice at 70 and 90 dpi. The normalized expression levels of p38γ, Lyn, RSK1, CaMK4β, nNOS, CREB, and PSD-95 (A) or levels of phosphorylated p38 (T180/Y182), Lyn (Y396), RSK1 (S380), nNOS (S847), CaMK4β (T196), and CREB (S133) (B) in the subcortical and cortical regions of each of the three scrapie-infected mice each time point shown by color bars. The proteins in dashed lines were not analyzed. (PPT 1 MB)

Additional file 3: Figure S2: Lower levels of MST1 and FOXO3 are phosphorylated to higher levels in the subcortical and cortical regions of scrapie-infected mice at 130 dpi. The normalized expression levels of DLK, MKK7, JNK2, MST1 and FOXO3 (A) or levels of phosphorylated JNK (T183/Y185), MST1 (T183), FOXO3 (S208), and cleaved MST1 (B) in the subcortical and cortical regions of each of the three scrapie-infected mice at each time point shown by color bars. The proteins boxed in dashed lines were not analyzed. (PPT 832 KB)
