# Supplementary material for: Activation of Pro-survival CaMK4β/CREB and Pro-death MST1 signaling at early and late times during a mouse model of prion disease
Source: Virol J. 2014 Sep 2;11:160. doi: 10.1186/1743-422X-11-160 (PMC4168054; doi:10.1186/1743-422X-11-160)
Supplement: Supplementary file 2 — Additional file 2: Figure S1: CREB is expressed and phosphorylated to higher levels in the subcortical and cortical regions of scrapie- than mock-infected mice at 70 and 90 dpi. The normalized expression levels of p38γ, Lyn, RSK1, CaMK4β, nNOS, CREB, and PSD-95 (A) or levels of phosphorylated p38 (T180/Y182), Lyn (Y396), RSK1 (S380), nNOS (S847), CaMK4β (T196), and CREB (S133) (B) in the subcortical and cortical regions of each of the three scrapie-infected mice each time point shown by color bars. The proteins in dashed lines were not analyzed. (PPT 1 MB) [file 12985_2014_2488_MOESM2_ESM.ppt]

## Slide 1
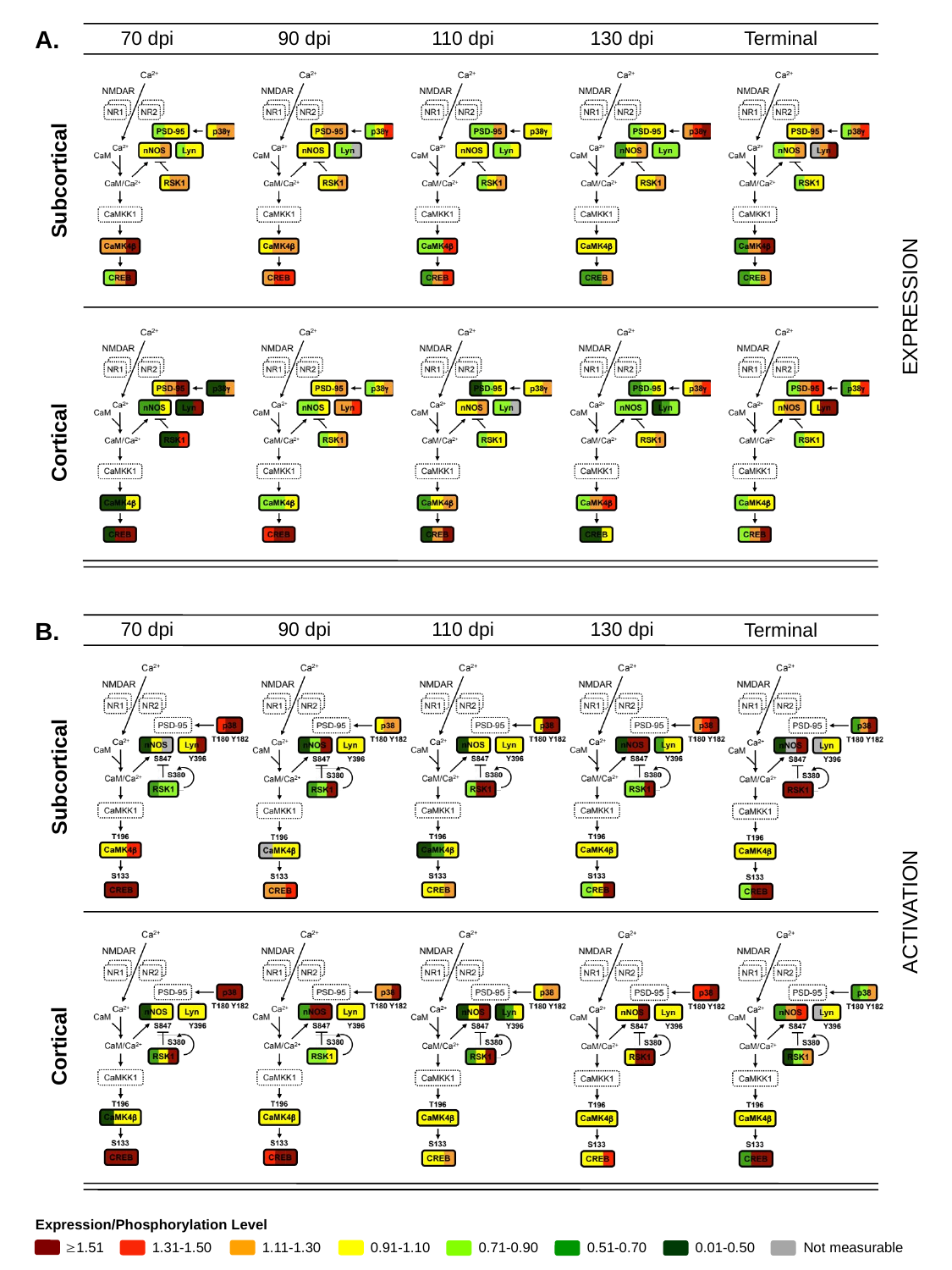

A.
70 dpi
90 dpi
110 dpi
130 dpi
Terminal
Subcortical
EXPRESSION
Cortical
B.
70 dpi
90 dpi
110 dpi
130 dpi
Terminal
Subcortical
ACTIVATION
Cortical
Expression/Phosphorylation Level
≥1.51
1.31-1.50
1.11-1.30
0.91-1.10
0.71-0.90
0.51-0.70
0.01-0.50
Not measurable

## Slide 2
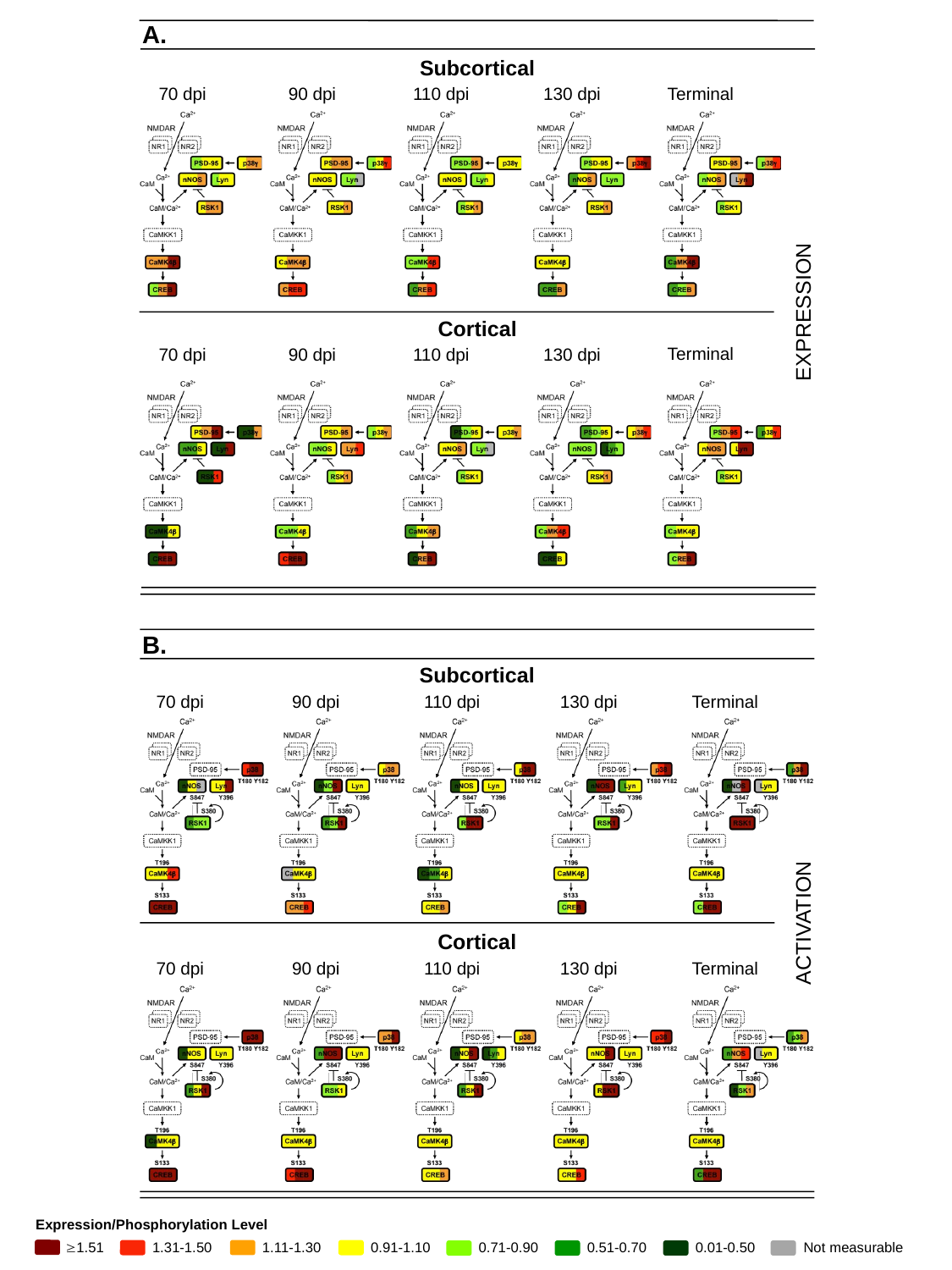

A.
Subcortical
70 dpi
90 dpi
110 dpi
130 dpi
Terminal
EXPRESSION
Cortical
Terminal
70 dpi
90 dpi
110 dpi
130 dpi
B.
Subcortical
Terminal
70 dpi
90 dpi
110 dpi
130 dpi
ACTIVATION
Cortical
Terminal
70 dpi
90 dpi
110 dpi
130 dpi
Expression/Phosphorylation Level
≥1.51
1.31-1.50
1.11-1.30
0.91-1.10
0.71-0.90
0.51-0.70
0.01-0.50
Not measurable
