# Supplementary material for: Activation of Pro-survival CaMK4β/CREB and Pro-death MST1 signaling at early and late times during a mouse model of prion disease
Source: Virol J. 2014 Sep 2;11:160. doi: 10.1186/1743-422X-11-160 (PMC4168054; doi:10.1186/1743-422X-11-160)
Supplement: Supplementary file 3 — Additional file 3: Figure S2: Lower levels of MST1 and FOXO3 are phosphorylated to higher levels in the subcortical and cortical regions of scrapie-infected mice at 130 dpi. The normalized expression levels of DLK, MKK7, JNK2, MST1 and FOXO3 (A) or levels of phosphorylated JNK (T183/Y185), MST1 (T183), FOXO3 (S208), and cleaved MST1 (B) in the subcortical and cortical regions of each of the three scrapie-infected mice at each time point shown by color bars. The proteins boxed in dashed lines were not analyzed. (PPT 832 KB) [file 12985_2014_2488_MOESM3_ESM.ppt]

## Slide 1
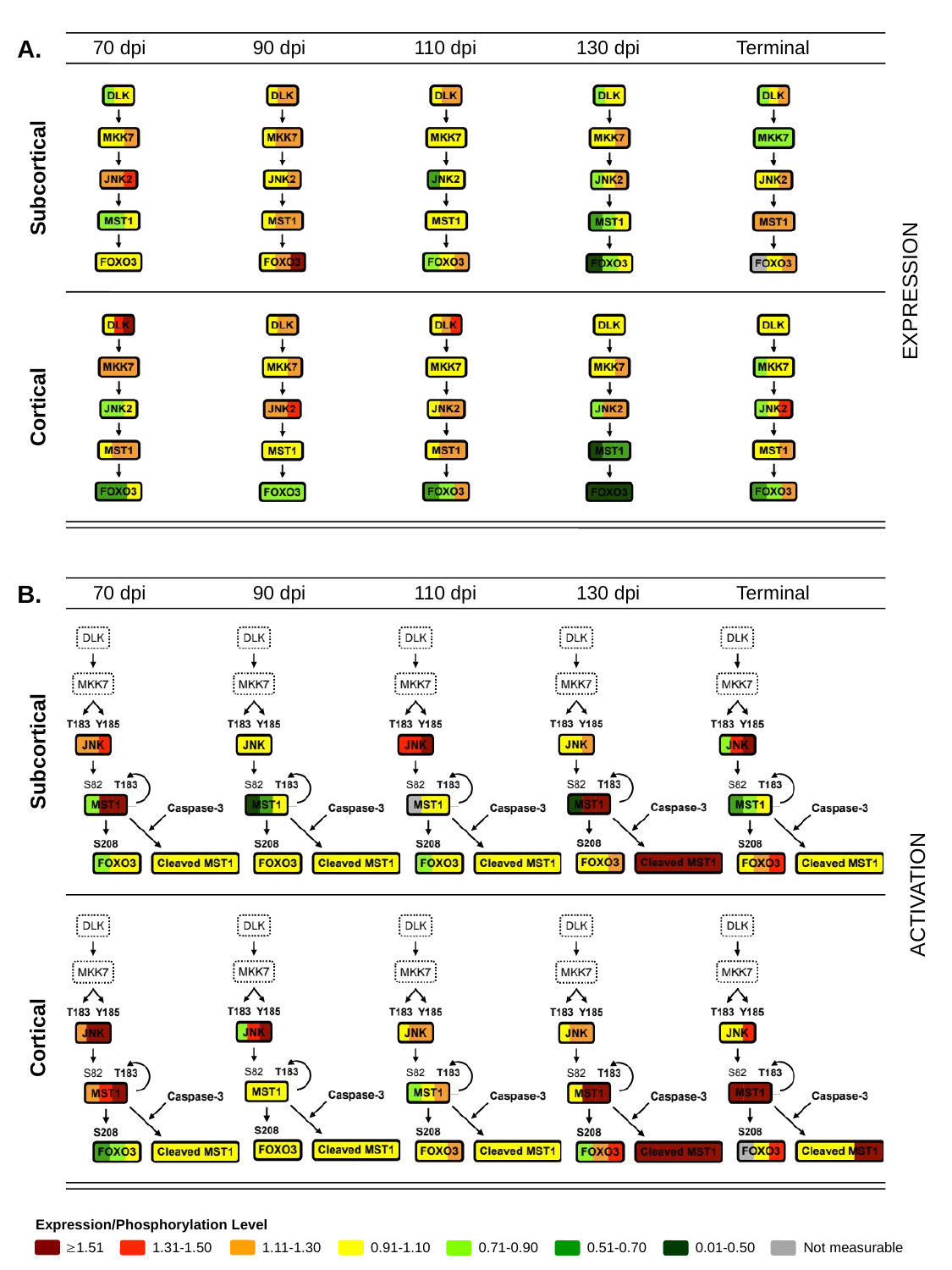

A.
70 dpi
90 dpi
110 dpi
130 dpi
Terminal
Subcortical
EXPRESSION
Cortical
B.
70 dpi
90 dpi
110 dpi
130 dpi
Terminal
Subcortical
ACTIVATION
Cortical
Expression/Phosphorylation Level
≥1.51
1.31-1.50
1.11-1.30
0.91-1.10
0.71-0.90
0.51-0.70
0.01-0.50
Not measurable

## Slide 2
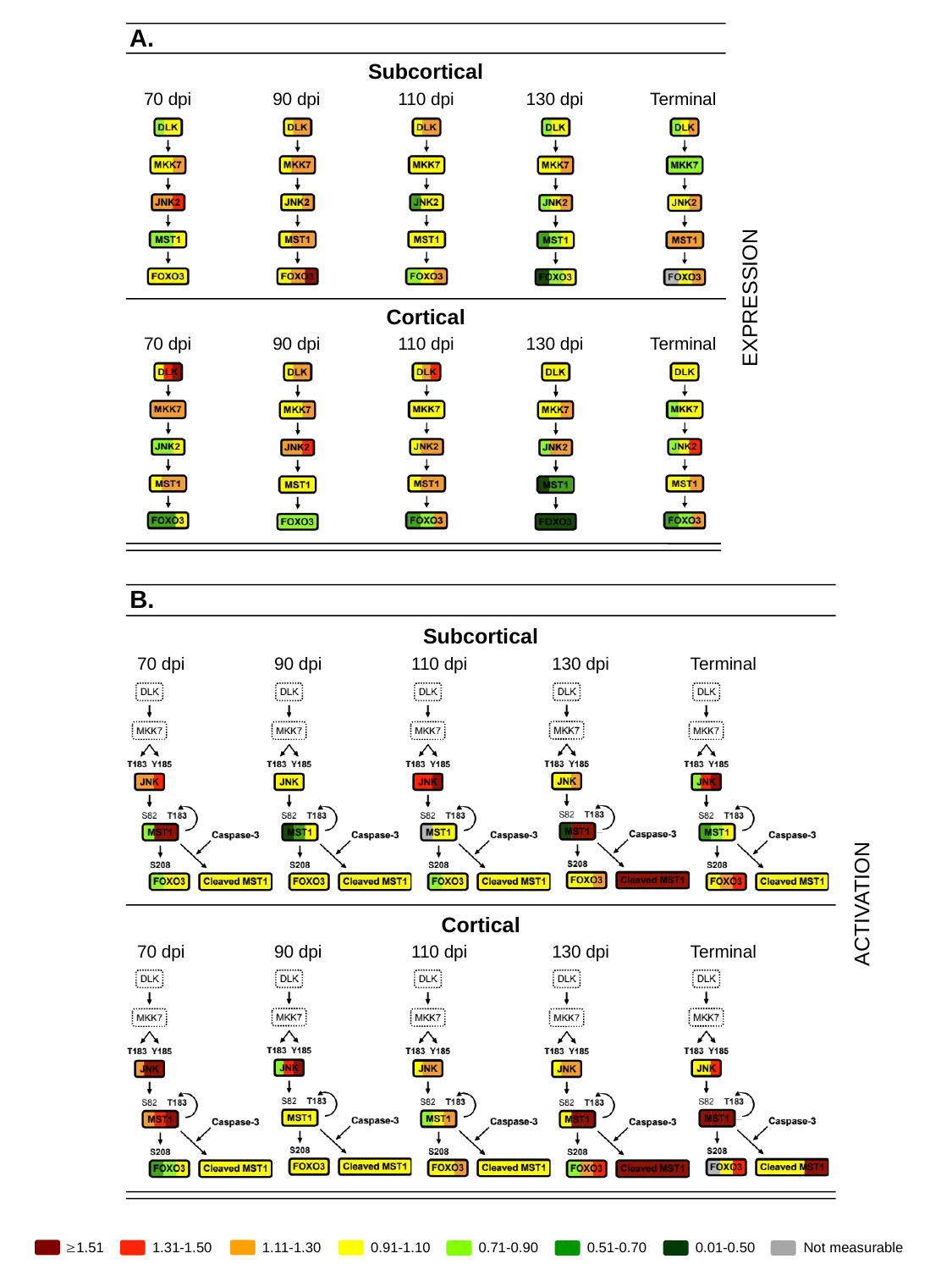

A.
Subcortical
70 dpi
90 dpi
110 dpi
130 dpi
Terminal
EXPRESSION
Cortical
70 dpi
90 dpi
110 dpi
130 dpi
Terminal
B.
Subcortical
70 dpi
90 dpi
110 dpi
130 dpi
Terminal
ACTIVATION
Cortical
70 dpi
90 dpi
110 dpi
130 dpi
Terminal
≥1.51
1.31-1.50
1.11-1.30
0.91-1.10
0.71-0.90
0.51-0.70
0.01-0.50
Not measurable
